# The potential role for prolactin-inducible protein (PIP) as a marker of human breast cancer micrometastasis

**DOI:** 10.1038/sj.bjc.6690799

**Published:** 1999-11

**Authors:** J W Clark, L Snell, R P C Shiu, F W Orr, N Maitre, C P H Vary, D J Cole, P H Watson

**Affiliations:** Department of Pathology and Physiology, University of Manitoba, Faculty of Medicine, D212-770 Bannatyne Avenue, Winnipeg, Manitoba, R3E 0W3, Canada; Department of Surgery, Center for Molecular Biology, Medical University of South Carolina, 171 Ashley Ave, Charleston, SC 29425, USA

**Keywords:** breast cancer, micrometastases, reverse transcription polymerase chain reaction, prolaction inducible protein, genetic marker

## Abstract

The prolactin-inducible protein (PIP/GCPD15) is believed to originate from a limited set of tissues, including breast and salivary glands, and has been applied as a clinical marker for the diagnosis of metastatic tumours of unknown origin. We have investigated the potential role of PIP mRNA as a marker of human breast cancer metastasis. Using reverse transcription polymerase chain reaction and Southern or dot blot analysis, PIP mRNA was detected in 4/6 breast cell lines, independent of oestrogen receptor (ER) status. In breast primary tumours (*n* = 97), analysed from histologically characterized sections, PIP mRNA was detected in most cases. Higher PIP mRNA levels correlated with ER^+^ (*P* = 0.0004), progesterone receptor positive (PR^+^) (*P* = 0.0167), low-grade (*P* = 0.0195) tumours, and also PIP protein levels assessed by immunohistochemistry (*n* = 19, *P* = 0.0319). PIP mRNA expression was also detectable in 11/16 (69%) of axillary node metastases. PIP mRNA expression, however, was also detected in normal breast duct epithelium, skin, salivary gland and peripheral blood leucocyte samples from normal individuals. We conclude that PIP mRNA is frequently expressed in both primary human breast tumours and nodal metastases. However, the presence of PIP expression in skin creates a potential source of contamination in venepuncture samples that should be considered in its application as a marker for breast tumour micrometastases. © 1999 Cancer Research Campaign


*British Journal of Cancer* (1999) **81**(6), 1002–1008
© 1999 Cancer Research Campaign
Article no. bjoc.1999.0799

### The potential role for prolactin-inducible protein (PIP) as a marker of human breast cancer micrometastasis

**JW Clark[1], L Snell[1], RPC Shiu[2], FW Orr[1], N Maitre[3], CPH Vary[4], DJ Cole[3] and PH Watson[1]**

Department of [1]Pathology and [2]Physiology, D212-770 Bannatyne Avenue, University of Manitoba, Faculty of Medicine, Winnipeg, Manitoba, Canada, R3E 0W3; [3]Department of Surgery and [4]Center for Molecular Biology, Medical University of South Carolina, 171 Ashley Ave, Charleston, SC 29425, USA

**Summary** The prolactin-inducible protein (PIP/GCPD15) is believed to originate from a limited set of tissues, including breast and salivary glands, and has been applied as a clinical marker for the diagnosis of metastatic tumours of unknown origin. We have investigated the potential role of PIP mRNA as a marker of human breast cancer metastasis. Using reverse transcription polymerase chain reaction and Southern or dot blot analysis, PIP mRNA was detected in 4/6 breast cell lines, independent of oestrogen receptor (ER) status. In breast primary tumours ($n$ = 97), analysed from histologically characterized sections, PIP mRNA was detected in most cases. Higher PIP mRNA levels correlated with $ER^+$ ($P$ = 0.0004), progesterone receptor positive ($PR^+$) ($P$ = 0.0167), low-grade ($P$ = 0.0195) tumours, and also PIP protein levels assessed by immunohistochemistry ($n$ = 19, $P$ = 0.0319). PIP mRNA expression was also detectable in 11/16 (69%) of axillary node metastases. PIP mRNA expression, however, was also detected in normal breast duct epithelium, skin, salivary gland and peripheral blood leucocyte samples from normal individuals. We conclude that PIP mRNA is frequently expressed in both primary human breast tumours and nodal metastases. However, the presence of PIP expression in skin creates a potential source of contamination in venepuncture samples that should be considered in its application as a marker for breast tumour micrometastases. © 1999 Cancer Research Campaign

**Keywords**: breast cancer; micrometastases; reverse transcription polymerase chain reaction; prolaction inducible protein; genetic marker

Detection of breast cancer micrometastases based on specific genetic markers may provide useful information to guide early therapeutic decisions. Immunohistochemical (IHC) and reverse transcription polymerase chain reaction (RT-PCR) methods offer the potential of improved sensitivity for detection of micrometastatic carcinoma cells that are missed by conventional histopathological examination (Raj et al, 1998; Pelkey et al, 1996; Lockett et al, 1998). Various biological markers have been proposed for the detection of breast cancer cells using these techniques, including keratin 19, muc1, EMA, CEA, HCG (Hoon et al, 1996; Tsuchiya et al, 1996; Mori et al, 1996; Schoenfeld et al, 1997). However, the frequency of expression of these markers is often related to tumour differentiation and is not always confined to breast tissue (Zippelius et al, 1997). Another promising breast specific marker is prolactin inducible protein (PIP) which is also known as gross cystic disease fluid protein-15 (GCDFP-15) (Haagensen et al, 1990; Wick et al, 1989; Murphy et al, 1987). IHC studies have previously shown that PIP is frequently expressed in human breast carcinomas and is comparatively specific for breast cancer (Wick et al, 1998; Wick et al, 1989; Mazoujian et al, 1983). While PIP expression is found to occur in tumours arising from skin and salivary gland, distinction from breast cancer is rarely a clinical issue and PIP protein has already found practical application as a marker for the recognition of breast origin of metastatic tumours (de Almeida & Pestana 1992; Fiel et al, 1996; Ormsby et al, 1995; Monteagudo et al, 1991). However, PIP is a secreted protein that is readily detectable in benign breast cyst fluid and plasma, which may impact on the significance of IHC detection of the protein (Haagensen et al, 1990). We have recently used PIP alongside other markers to explore its value in detection of breast micrometastases (Lockett et al, 1998a; Lockett et al, 1998b), however, the incidence and pattern of PIP expression at the level of RT-PCR is not known. Our purpose in this study was to evaluate the potential of PIP mRNA as a marker for the detection of breast cancer cells by assessing the frequency of PIP mRNA expression in human breast cell lines and in breast tumors in relation to tissue composition and pathology.

*Received 10 December 1998*
*Revised 12 May 1999*
*Accepted 13 May 1999*

*Correspondence to:* PH Watson

### MATERIALS AND METHODS

### Human cell lines

The human breast cancer cell lines (T47D, ZR 75, MDA-MB-231, BT 474 and MCF-7) and the normal human breast cell line, HBL-100, were obtained from the American Type Culture Collection (ATCC, Rockville, MD, USA). All cell lines were cultured as described previously in Dulbecco's modified Eagle's medium (DMEM) containing 10% fetal bovine serum (FBS), 1% 1 mg $ml^{-1}$ insulin, 1% 35% (w/v) glucose, 1% penicillin–streptomycin and 1% L-glutamine. Cells were harvested with 5% trypsin (v/v) from culture flasks.

### Human tissue samples

A cohort of 97 primary breast tumour samples was obtained from the Manitoba Breast Tumor Bank located in the Department of Pathology, Faculty of Medicine, University of Manitoba. The cohort was selected initially on the basis of oestrogen receptor

(ER) status so as to ensure a wide range of ER levels to determine any correlation between ER status and PIP expression. The tumour bank collected all breast tumour specimens on ice which were then bisected to provide mirror image tissues for formalin-fixed, paraffin-embedded blocks and matching frozen tissue blocks stored at –70°C (Hiller et al, 1996). The pathological and histological parameters (including tumour type, grade, invasive and normal cell content) were then assessed uniformly by one pathologist in sections from the paraffin block and entered into a database enabling selection by specific criteria (Watson, 1996). Tumour grading was performed using the Nottingham system (Elston & Ellis, 1991) and steroid receptor levels were measured by ligand binding assay performed on an adjacent piece of tumour tissue. ER and progesterone receptor (PR) values above 3 fmol $mg^{-1}$ and 15 fmol $mg^{-1}$ total protein respectively were deemed positive. A second cohort of five primary tumours was also selected from the tumour bank on the basis of association with matching frozen tissue in the bank from a synchronous nodal metastasis.

A third cohort of axillary lymph node samples from 32 patients with metastatic breast tumours undergoing breast cancer surgery was obtained from the Department of Surgery at the Medical University of South Carolina. These samples were collected prospectively from patients with primary tumours associated with a range of clinical stages (12 stage I, 12 stage II, eight stage III) and included 16 samples from patients who were histologically node-positive and 16 from patients who were node-negative. Immediately after resection, the axillary lymph node specimens were identified and separated from the specimen by a pathologist. All lymph nodes > 1 cm were bisected, with half of the node sent for routine histological evaluation and the other half for RT-PCR screening. The RT-PCR screened lymph nodes were snap frozen at –70°C until being processed to extract total RNA.

Normal tissue samples from several potential sites of breast tumour metastasis were also obtained from the Manitoba Breast Tumor Bank. Normal human peripheral blood lymphocytes (PBL) were isolated from 5 ml blood samples drawn from each of 11 healthy volunteers (females and males, 22–29 years old). The red blood cells in each 5 ml samples were lysed by adding 25 ml of lysing reagent (140 mM $NH_4Cl_2$ and 17 mM Tris, pH 7.6) and leaving the mixture to incubate for 10 min at 37°C. This was followed by centrifugation at 2000 rpm for 10 min to pellet white blood cells, removal of the supernatant and re-suspension of PBLs in PBS.

### Sensitivity assay

To determine the sensitivity of the RT-PCR assay, T47D human breast cancer cells ($PIP^+$) were diluted into a background of MDA-MB-231 cells ($PIP^-$) so as to obtain a range of concentrations of $PIP^+$ cells from 1 to 1000 cells in $10^6$ $PIP^-$ cells prior to RNA extraction and RT-PCR assay. Cell numbers were determined by directly counting aliquots of cells in suspension under a microscope using a haemocytometer.

### RNA extraction and RT-PCR

Total RNA from all tumour, tissue and cell pellet samples was isolated using similar commercial extraction reagents, either Tri-reagent or Tri-zol reagents and protocols according to the manufacturer's instructions (Molecular Research Center Inc, Cincinnati, OH, USA and BRL). RNA samples were quantified by performing spectrophotometry. Samples were presumed to be free of DNA and proteins if the OD 260/280 ratio was 1.6–1.8. All RNA samples were stored at –70°C until further use.

Reverse transcription of mRNA to cDNA was performed as previously described (Hiller et al, 1996) with the following modifications. All PCR primers were designed to cross intron–exon boundaries. The PIP primers were sense (5′-GCTCAGGACAA-CACTCGGAA-3′) and antisense (5′-ATAACATCAACGACG-GCTGC-3′) corresponding to positions 107 and 356 of the cDNA sequence (Murphy et al, 1987), and GAPDH primers were sense (5′-ACCCACTCCTCCACCTTTG-3′) and antisense (5′-CTCT-TGGCTCTTGCTGGG-3′) (Ercolani et al, 1988). Preliminary experiments were performed with cell line and tumour RNA samples to establish the appropriate RNA input and PCR cycle number conditions to achieve amplification with both PIP and gluceraldehyde 3-phosphate dehydrogenase (GAPDH) primers in the linear range in a typical sample. Amplification of GAPDH or actin was then performed in duplicate samples, for every experimental sample, to provide an internal indicator as to the quality of the cDNA of each sample. The PCR consists of an initial 5-min preheating step at 94°C, followed by repeated cycles of a 1-min denaturing step at 94°C, a 1-min primer annealing step at 54°C, and a 90 s elongation step at 72°C. Cycle numbers to achieve amplification in the linear range were 40 for PIP and 35 for GAPDH and actin. Once thermal cycling was completed, samples underwent one final elongation step at 72°C for 7 min. Tumours were processed in batches of 12 samples, from frozen sectioning to RNA extraction, RT in triplicate and PCR. For each batch controls included RT- and RNA-controls, and both $PIP^+$ (T47D) and $PIP^-$ (MDA-MB-231) RNA controls. All primary tumour PCR signals were assessed in gels and autoradiographs by video image capture and computer analysis using MCID-M4 Imaging Research Inc, version 2.0 image analysis program. PIP expression was standardized to GAPDH expression assessed in separate PCR reactions from the same RT reaction and run in parallel on the same gel and then expressed relative to the levels in the T47D cell line standard. To correct for any differences in processing between gels PIP levels were further standardized to a set of PCR product standards incorporated into each gel.

### Southern and dot blot analysis

For Southern blot analysis following PCR amplification, PCR products were loaded into a 1% agarose gel. After electrophoresis the gel was exposed to ethidium bromide, illuminated with ultraviolet light and photographed. For hybridization, gels were denatured in 0.5 M sodium hydroxide (NaOH), 1 M sodium chloride (NaCl) for 30 min at room temperature and neutralized for 30 min in 1.5 M Tris–HCl pH 7.4, 3 M NaCl. PCR product cDNA was then transferred to Zeta-Probe membrane according to the Zeta-Probe protocol (Bio-Rad Laboratories, Hercules, CA, USA) and membranes were then dried in an 80°C oven for 30 min. For probing membranes were prehybridized with 10 ml of hybridization solution (50 ml of formamide, 12 ml of 1 M $Na_2HPO_4$, 5 ml of 5 M NaCl, 7 g of sodium dodecyl sulphate (SDS), and 200 µl of 0.5 M EDTA in 100 ml with $ddH_2O$) at 42°C for 2 h with agitation. Hybridization was then conducted at 42°C for 24 h with a hPIP cDNA probe (Murphy et al, 1987) $^{32}P$ labelled by the random priming method and purified with a NICK chromatography column (Pharmacia Biotech, Inc.). Hybridization membranes were washed at room temperature for 1 h in a solution of 2 × SSC

© 1999 Cancer Research Campaign

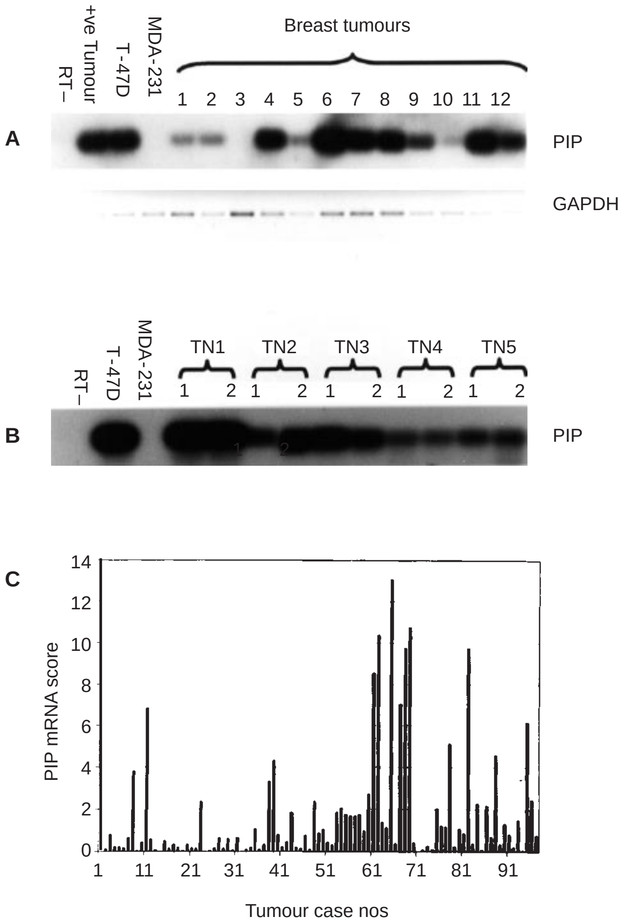

**Figure 1** PIP mRNA expression in breast tumours. The upper panels show a representative set of 12 primary tumours (**A**) and a set of five cases (**B**, TN 1–5) comprising primary breast tumours (1) and their corresponding nodal metastases (2) analysed for PIP mRNA levels by RT-PCR – Southern blot. Corresponding GAPDH levels, determined as described in Materials and Methods relative to T47D cells and a reference tumour (positive controls) and MDA-MB-231 cells (negative control) are also shown. T47D RNA subjected to reverse transcription reaction without RT-enzyme and subsequent PCR is also shown (RT-control). In the lower panel the chart shows a graphical representation of the level of PIP mRNA expression in tumours relative to ER status (**C**). Tumour case numbers 1–30 are ER– (< 3 fmol $mg^{-1}$ protein), 31–49 are ER low positive (3–20 fmol $mg^{-1}$ protein) and 50–97 are ER high positive (> 20 fmol $mg^{-1}$ protein)

(standard saline citrate) and 0.1% SDS, then 1 h in 0.5 × SSC and 0.1% SDS, and 1 h in a 65°C waterbath in 0.1 × SSC and 0.1% SDS. Bands were then visualized after autoradiography for 2–6 h.

For dot blot analysis, 1 µl of each PCR reaction sample was deposited on a strip of Sure Blot Hybridization Membrane (Oncor, Gaithersburg, MD, USA) and left to dry for 5 min. The membrane was incubated for 5 min in a solution of 0.2 M NaOH at room temperature after which the strip was incubated for another 30 min at 56°C in Blocking Buffer (0.2% I-Block; Tropix, Bedford, MA, USA), 1 × PBS, 0.5% SDS). The same PIP cDNA probe (Murphy et al, 1987) was labelled using alkaline phosphatase as previously described (Vary et al, 1996) and then added to the same tube at a 1:3000 dilution of stock (50 ng $ml^{-1}$) and hybridized for 15 min. The membrane was then washed 3 times for 10 min in Wash Buffer (10 × PBS, 0.5% SDS) and twice in AMPPD Buffer (1 mM magnesium chloride hexahydrate, 0.1 M diethanolamine, pH 10). Finally, the membrane was incubated 30 min in the dark with 1% CSPD (Tropix, Bedford, MA, USA) in AMPPD buffer. Dots were visualized by exposure to 4× autoradiography film for 10 min.

**Table 1** Relationship between mean PIP mRNA levels in primary breast tumours and prognostic parameters

| | | *n* | Mean | (s.d.) | *P*-value |
|---|---|---|---|---|---|
| ER | –ve | 30 | 0.69 | (1.4) | 0.0004 |
| | +ve | 67 | 2.12 | (3) | |
| PR | –ve | 52 | 1.04 | (1.8) | 0.0167 |
| | +ve | 45 | 2.42 | (3.3) | |
| Grade | low | 16 | 2.59 | (3.5) | |
| | mod | 41 | 1.83 | (2.9) | 0.0195 |
| | high | 40 | 1.16 | (1.9) | |
| Nodal status | +ve | 37 | 2.09 | (3.3) | NS |
| | –ve | 42 | 1.18 | (1.8) | |
| | unknown | 18 | | | |
| Size | <2 cm | 14 | 2.39 | (4.5) | |
| | 2–5 cm | 45 | 1.36 | (2.1) | NS |
| | > 5 cm | 16 | 1.33 | (1.9) | |
| | Unknown | 22 | | | |

PIP mRNA score (mean and s.d.) shown was derived as described in Materials and Methods. *P*-values correspond to Spearman correlation test

**Table 2** PIP RT-PCR screening of axillary lymph nodes compared to routine histopathology

| | | Pathology | | |
|---|---|---|---|---|
| | | +ve | –ve | Total |
| RT-PCR | +ve | 11 (69%) | 6 (37%) | 17 (53%) |
| | –ve | 5 (31%) | 10 (63%) | 15 (47%) |
| | Total | 16 (50%) | 16 (50%) | 32 (100%) |

### Immunohistochemistry and in situ hybridization

Immunohistochemical detection of PIP expression was performed using a commercially available monoclonal antibody (Signet Laboratories Inc, Dedham, MA, USA) and protocol as recommended. PIP was assessed in paraffin sections from a subset of 19 primary tumours, selected to correspond to a wide range of PIP mRNA levels as determined by the RT-PCR assay based on frozen tissue sections from the same cases. PIP protein was scored by estimating the average signal intensity (on a scale of 0–3) and the proportion of cells showing a positive signal and scored as 0 (none), 0.1 (less than one-tenth), 0.5 (less than one-half), or 1.0 (greater than one-half). The intensity and proportion scores were then multiplied to give an overall score. In situ hybridization was performed as previously described (Leygue et al, 1996) on 5-µm paraffin sections from normal and tumour tissue with both sense and antisense PIP riboprobes synthesized using UTP ($^{35}S$) to label the probes using Riboprobe[R] Systems (Promega, Madison, WI, USA) according to the manufacturer's instructions.

### RESULTS

### Analysis of sensitivity and PIP expression in cell lines

Multiple experiments were performed to determine the threshold for detection of $PIP^{+}$ cells in a background of $PIP^{-}$ cells, using the

 © 1999 Cancer Research Campaign

**Table 3** Correlation between PIP protein and mRNA expression in a subset of 19 tumours

| Case no. | Int % | PIP IHC | PIP RT-PCR |
|---|---|---|---|
| 11913 | [0 × 0] | 0 | 0.023 |
| 11365 | [1 × 0.1] | 0.1 | 0.030 |
| 11657 | [1 × 0.1] | 0.1 | 0.048 |
| 10927 | [1 × 0.5] | 0.5 | 0.073 |
| 10970 | [0 × 0] | 0 | 0.170 |
| 11097 | [2 × 0.5] | 1 | 0.205 |
| 11836 | [1 × 0.1] | 0.1 | 0.350 |
| 11909 | [2 × 1] | 2 | 0.415 |
| 11526 | [1 × 1] | 1 | 0.469 |
| 10975 | [2 × 1] | 2 | 0.748 |
| 11341 | [2 × 1] | 2 | 0.964 |
| 11729 | [1 × 0.1] | 0.1 | 1.180 |
| 11339 | [0 × 0] | 0 | 1.840 |
| 11603 | [3 × 0.1] | 0.3 | 2.020 |
| 11288 | [1 × 0.5] | 0.5 | 2.470 |
| 11010 | [2 × 1] | 2 | 2.718 |
| 11903 | [2 × 0.5] | 1 | 4.320 |
| 11734 | [1 × 1] | 1 | 4.610 |
| 11152 | [2 × 1] | 2 | 6.190 |

Int = intensity, % = percentage of positive staining cells, PIP IHC = PIP protein score derived as described in Materials and Methods from the product of intensity and proportion of positive cells by immunohistochemistry assay, PIP RT-PCR = PIP mRNA score as described in Materials and Methods.

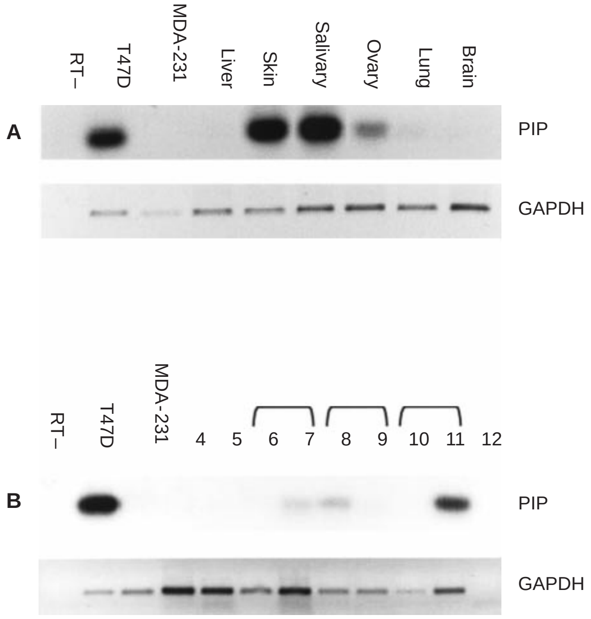

**Figure 2** PIP mRNA expression in normal tissues. The upper panel shows PIP levels in a set of normal tissues that are sites of breast cancer metastasis (**A**) and the lower panel shows normal peripheral blood lymphocytes isolated from venepuncture samples from five individuals (**B**). Lanes are as follows: lane 4 normal male; lane 5 normal female, lanes 6 & 7, 8 & 9 and 10 & 11 are from three normal females on two separate occasions each, lane 12, RNA minus PCR control

RT-PCR/Southern blot assay. In different experiments the detection limit varied between 10 and 50 PIP$^+$ cells in a background of $1 \times 10^6$ PIP$^-$ cells in different experiments (data not shown).

Of the six human breast cell lines (T47D, ZR 75, BT 474, MCF-7, MDA-MB-231 and HBL-100) that were analysed, four showed positive expression of PIP mRNA (data not shown). The rank order of expression amongst the PIP$^+$ cell lines was: T47D > ZR 75 > HBL-100 > BT 474.

### Analysis of PIP mRNA expression in human breast tumours

PIP mRNA expression in 97 primary tumours was assessed by three independent RT and PCR reactions and expressed as a PIP mRNA score. This was calculated from the mean intensity of PIP mRNA signals for each tumour, standardized to the GAPDH signal from three separate PCR reactions performed on the same RT reactions and then standardized to the reference PIP signal as determined in the T47D cell line (Figure 1). PIP was negative or very low (< 5% of the T47D level) in eight tumours (8% cases). Amongst the PIP$^+$ tumours, 37 (38% cases) expressed PIP at levels between 5% and 50% of that of the T47D cell, 30 (31% of cases) expressed PIP at levels that were similar, between 50% and 200%, and 22 (23% of cases) expressed PIP at higher levels. Further analysis of PIP expression levels in relation to clinical-pathological factors found a significant correlation between higher levels of PIP expression in the primary tumours and higher ER ($P = 0.0004$, $r = 0.32$) and PR levels ($P = 0.0167$, $r = 0.24$) and lower Nottingham tumour grade score ($P = 0.0195$, $r = 0.24$, Spearman correlation test). Similar analysis of these same parameters as discontinuous variables was performed and confirmed these associations. Mean (s.d.) PIP mRNA levels were higher in ER$^+$ ($n = 67$, $2.12^{(3.01)}$) versus ER$^-$ ($n = 30$, $0.69^{(1.39)}$) tumours ($P = 0.0004$ Mann–Whitney test), PR$^+$ ($n = 45$, $2.42^{(3.33)}$) versus PR$^-$ ($n = 52$, $1.04^{(1.78)}$ tumours ($P = 0.001$). PIP mRNA levels also increased from well differentiated to poorly differentiated tumours when assessed as three grade categories, although these differences fell short of achieving statistical significance (low-grade, $n = 16$, $2.59^{(3.49)}$, moderate-grade, $n = 41$, $1.83^{(2.92)}$, high-grade, $n = 40$, $1.15^{(1.95)}$, $P = 0.099$ ANOVA test). No relationship was seen between PIP mRNA expression and tumour size or nodal status (Table 1).

In five additional cases with matching primary and nodal metastasis tissue, analysis confirmed that PIP expression is conserved at similar levels between primary and metastatic cells (Figure 1B). Furthermore, RT-PCR analysis of lymph nodes from three patients undergoing elective carotid endarterectomy without any current or prior history of cancer was negative. Detection of PIP mRNA expression was also performed on axillary lymph nodes from 32 different patients using the same RT-PCR assay but with minor modifications to the method of detection. This assay used the same PCR assay and primers and PIP cDNA probe, but detection was performed by use of a non-radioactive alkaline phosphatase labelling method for the probe applied to a dot blot for detection of the PCR product. Overall PIP mRNA was detected in 17/32 lymph nodes (53%) and increasing PIP positivity reflected the tumour stage with 2/12 (17%) stage I cases positive compared to 9/12 (75%) stage II and 6/8 (75%) of stage III cases positive by RT-PCR assay. Amongst the subset of cases that were positive by histology, 11/16 (69%) were also positive by PIP RT-PCR and 5/16 (31%) were negative for PIPmRNA. Amongst the lymph nodes that were negative by histology, 6/16 (37%) were PIP RT-PCR positive (Table 2).

### Immunohistochemistry and in situ hybridization

PIP mRNA levels determined by RT-PCR assay was compared to protein levels assayed in a subset of 19 cases by immunohisto-

© 1999 Cancer Research Campaign 

chemistry (Table 3). PIP mRNA level correlated well with protein level ($r$ = 0.493, $P$ = 0.0319, Spearman test) and using cut-off points of < 5% PIPmRNA score and < 0.1 for PIP IHC score there was also 89% concordance. Additional study by in situ hybridization was performed on one tumour and one normal breast tissue which confirmed previous observations that PIP mRNA expression was confined to epithelial cells but showed that PIP mRNA is also expressed by both normal and neoplastic epithelial cells (data not shown).

### Analysis of PIP expression in normal human tissues

PIP mRNA was expressed at comparable levels to the T47D breast tumour cell in several normal tissues examined including skin salivary gland and ovary (Figure 2A). Very low levels of PIP expression were observed in lung, whereas brain and liver were negative. Immunohistochemistry analysis of skin demonstrated that PIP protein expression was confined to sweat gland-like structures in the dermis. Also mRNA analysis of PBLs from 11 normal people that were analysed, all were PIP$^-$ on at least one occasion (Figure 2B). Repeat samples from three individuals showed positive signals on other occasions that could not be explained as systematic contamination at the RT-PCR step.

### DISCUSSION

We have shown that PIP mRNA is frequently expressed by primary breast tumours, although higher levels of expression occur in well-differentiated and ER/PR$^+$ tumours. Nevertheless, PIP mRNA expression is also often conserved within the corresponding lymph node metastases. Given confirmation of the relative specificity for breast tissue that has also previously been established at the protein level by immunohistochemical studies (Wick et al, 1989), it is clear that PIP mRNA expression is a potential marker for breast micrometastasis. The presence of occasional positive signals in morphologically normal breast epithelium and in peripheral blood samples from normal individuals also indicates that, in common with most other markers, there is a need for caution in the application of PIP as a single marker for metastatic disease.

Many recent studies have concentrated on either IHC or RT-PCR assays to detect specific markers that may indicate the presence of micrometastatic disease. It is clear, however, that several pitfalls need to be considered in the practical application of individual markers (Dingemans et al, 1997). For example, while IHC assay allows morphological confirmation of the origin of positive signals it may detect some secreted proteins beyond the context of the known cell of origin. In contrast, RT-PCR may be more sensitive but does not allow direct confirmation of a positive signal in the context of the appropriate cell morphology. RT-PCR assay can also face problems that could arise from the presence of pseudogene DNA sequences and low levels of background gene expression in target tissues (Bostick et al, 1998; Lopez-Guerrero et al, 1997; Zippelius et al 1997). Improvement in specificity, rather than sensitivity, is needed. Amongst the several markers that have been used for the detection of breast micrometastases, keratin 19 and muc1 have been the most widely used (Noguchi et al, 1996; McGuckin et al, 1996). Improvements in specificity might best be achieved through a combination of these with other markers (Min et al, 1998) and a composite of technical approaches to their detection, refined still further by an appreciation of the limitations of individual markers (Zippelius et al 1997).

This study is aimed at establishing the potential of PIP as a supplementary breast tumour cell marker. The GCDFP-15/PIP gene encodes a protein that is found in high concentrations in gross cystic disease of breast and in fluids of normal apocrine glands such as sweat, tears and seminal fluid (Haagensen et al, 1990). Given the low incidence and clinical distinctiveness of tumours arising from other source tissues, PIP/GCDFP-15 protein has already been considered as a breast cell specific marker, complementary to keratin (de Almeida & Pestana 1992). This potential is based on the fact that PIP expression can be detected by IHC in up to 76% of breast carcinomas (Wick et al, 1989) and there is a high degree of concordance between PIP expression in 1° primary carcinomas and nodal metastases (Mazoujian et al, 1989). Expression of this marker has been associated with apocrine differentiation, but there is not a direct concordance with Muc1 (Soomro & Shousha, 1992). While IHC, in situ hybridization and Northern analysis have all found expression at a similar frequency (Murphy et al, 1987; Pagani et al, 1994), the prevalence and specificity of PIP mRNA for breast cancer at the sensitivity level of RT-PCR has not been established. Using RT-PCR we have found expression of PIP mRNA is readily detectable in most human breast cell lines and breast tumours. This is more frequent than previous IHC studies, which have reported the proportion of PIP positivity between 55% and 72% (Wick et al, 1989; Mazoujian et al, 1983). Not only is RT-PCR recognized as a highly sensitive technique but it is clear that, in this study, the very high frequency of PIP mRNA expression could in some cases be partly attributable to weak signals that originated only within residual normal breast elements. Consistent with this conclusion is the fact that a minor component of histologically detectable normal epithelium was found to be present within some (15%) of the 97 cases studied, which also mostly showed low levels of PIP expression. We estimate therefore that the true frequency of PIP mRNA positive primary tumours is approximately 85% of cases. This is consistent with our data where PIP mRNA expression was detected in approximately 70% nodal metastases (Table 2) and that of others (Mazoujian et al, 1989) and the fact that PIP expression is often conserved between primary and nodal metastases (Figure 1B), as also documented by others (Mazoujian et al, 1989; Wick et al, 1998). The presence of PIP mRNA in lymph nodes that are histologically negative (on the basis of assessment of a single haematoxylin and eosin (H&E)-stained diagnostic section) may suggest the presence of occult metastases (Ferrari et al, 1997). Previous studies have found micrometastases up to 25% lymph nodes from breast cancer patients when this is pursued intensively by histology and immunohistochemistry applied to serial sections, (McGuckin et al, 1996) and in 15–25% of cases when RT-PCR analysis is applied using other tumour markers such as muc1 (Noguchi et al, 1994) or β-human chorionic gonadotrophin (Hoon et al 1996). More sensitive nested-RT-PCR assays to detect both prostate-specific antigen and prostate-specific membrane antigen in histologically negative lymph nodes from prostate cancer patients have found positive tumour marker expression in up to 79% of histologically negative cases. Nonetheless, as with other current markers, the possibility of ectopic expression within normal tissues in some patients remains to be ruled out (Ferrari et al 1997).

While normal PBL samples taken from healthy individuals were all usually negative, positive results were also obtained from

 © 1999 Cancer Research Campaign independent samples taken from the same individuals. It is already known that PIP protein is present at relatively high levels in some tissues other than breast, including skin, sweat glands and salivary gland (Viacava et al, 1998; Wick et al, 1998). It therefore seems possible that these false-positive results are attributable to contamination from PIP expressing cells from sweat glands in the skin, removed during venepuncture. If this interpretation is correct then this problem might be minimized in any similar future study of PIP by obtaining several blood samples at each venapuncture and retaining only the final sample for analysis (de Graaf et al, 1997).

In this uniformly assessed tumour cohort increased PIP mRNA expression was significantly associated with low-grade and ER and PR positivity, both features that could be interpreted to indicate either biological potential or cellular differentiation. However, while PIP was not correlated with other indicators of biological potential such as tumour size or nodal status, PIP expression has previously been associated with cellular differentiation. PIP mRNA in vitro in breast cells in culture is highest in well-differentiated $ER^+$ cells where PIP expression has also been shown to be influenced by steroid hormones (Murphy et al, 1987) and expression in non-neoplastic and neoplastic breast tissues in vivo has been associated with specific morphological features of apocrine cellular differentiation (Haagensen et al, 1990). It should be noted that while our results are in agreement with the trends seen in other recent studies (Hall et al, 1998; Bundred et al, 1990), previous IHC studies, several larger than the present one (Mazoujian et al, 1989; Wick et al, 1989), have not established an association with these parameters. However, levels of PIP protein in breast cancer tissue, unlike PIP mRNA, may be affected by the fact that PIP is a secreted protein that is present at high levels in breast duct secretions and the serum and so can originate from adjacent breast tissues or other tissue normal tissues (Haagensen et al, 1990; Manni et al, 1984).

We conclude that the PIP gene has potential as a marker of breast micrometastasis. This is supported by the following attributes: (1) PIP is expressed by most primary breast tumours, (2) expression is often conserved in nodal metastases, and (3) this gene is not expressed in several tissues that are often targets of breast tumour metastasis. In common with several other genes proposed as tumour markers, our results demonstrate the potential for false-negative and false-positive results. The impact of this on the clinical identification of true micrometastases should be recognized and strategies developed to minimize these errors through parallel assessment of unrelated tumour markers.

### ACKNOWLEDGEMENTS

This work was supported by grants from the Medical Research Council of Canada (MRC) and the Canadian Breast Cancer Research Initiative. The Manitoba Breast Tumor Bank is supported by funding from the National Cancer Institute of Canada (NCIC). PHW is an MRC Scientist.

### REFERENCES

Bostick PJ, Chatterjee S, Chi DD, Huynh KT, Giuliano AE, Cote R and Hoon DS (1998) Limitations of specific reverse-transcriptase polymerase chain reaction markers in the detection of metastases in the lymph nodes and blood of breast cancer patients. *J Clin Oncol* **16**: 2632–2640

Bundred NJ, Stewart HJ, Shaw DA, Forrest AP and Miller WR (1990) Relation between apocrine differentiation and receptor status, prognosis and hormonal response in breast cancer. *Eur J Cancer* **26**: 1145–117

de Almeida PC and Pestana CB (1992) Immunohistochemical markers in the identification of metastatic breast cancer. *Breast Cancer Res Treat* **21**: 201–210

de Graaf H, Maelandsmo GM, Ruud P, Forus A, Oyjord T, Fodstad O and Hovig E (1997) Ectopic expression of target genes may represent an inherent limitation of RT-PCR assays used for micrometastasis detection: studies on the epithelial glycoprotein gene EGP-2. *Int J Cancer* **72**: 191–196

Dingemans AM, Brakenhoff RH, Postmus PE and Giaccone G (1997) Detection of cytokeratin-19 transcripts by reverse transcriptase-polymerase chain reaction in lung cancer cell lines and blood of lung cancer patients [see comments]. *Lab Invest* **77**: 213–220

Elston CW and Ellis IO (1991) Pathological prognostic factors in breast cancer. I. The value of histological grade in breast cancer: experience from a large study with long-term follow-up. *Histopathology* **19**: 403–410

Ercolani L, Florence B, Denaro M and Alexander M (1988) Isolation and complete sequence of a functional human glyceraldehyde-3-phosphate dehydrogenase gene. *J Biol Chem* **263**: 15335–15341

Ferrari AC, Stone NN, Eyler JN, Gao M, Mandeli J, Unger P, Gallagher RE and Stock R (1997) Prospective analysis of prostate-specific markers in pelvic lymph nodes of patients with high-risk prostate cancer [see comments]. *J Natl Cancer Inst* **89**: 1498–1504

Fiel MI, Cernaianu G, Burstein DE and Batheja N (1996) Value of GCDFP-15 (BRST-2) as a specific immunocytochemical marker for breast carcinoma in cytologic specimens. *Acta Cytol* **40**: 637–641

Haagensen DE, Jr, Dilley WG, Mazoujian G and Wells SA, Jr (1990) Review of GCDFP-15. An apocrine marker protein. *Ann NY Acad Sci* **586**: 161–173

Hall RE, Clements JA, Birrell SN and Tilley WD (1998) Prostate-specific antigen and gross cystic disease fluid protein-15 are co-expressed in androgen receptor-positive breast tumours. *Br J Cancer* **78**: 360–365

Hiller T, Snell L and Watson PH (1996) Microdissection RT-PCR analysis of gene expression in pathologically defined frozen tissue sections. *Biotechniques* **21**: 38–40, 42, 44

Hoon DS, Sarantou T, Doi F, Chi DD, Kuo C, Conrad AJ, Schmid P, Turner R and Guiliano A (1996) Detection of metastatic breast cancer by beta-hCG polymerase chain reaction. *Int J Cancer* **69**: 369–374

Leygue E, Snell L, Hiller T, Dotzlaw H, Hole K, Murphy LC and Watson PH (1996) Differential expression of psoriasin messenger RNA between in situ and invasive human breast carcinoma [published erratum appears in Cancer Res 1997 Feb 15; 57(4): 793]. *Cancer Res* **56**: 4606–4609

Lockett MA, Baron PL, PH, OB, Elliott BM, Robison JG, Maitre N, Metcalf JS and Cole DJ (1998*a*) Detection of occult breast cancer micrometastases in axillary lymph nodes using a multimarker reverse transcriptase-polymerase chain reaction panel. *J Am Coll Surg* **187**: 9–16

Lockett MA, Metcalf JS, Baron PL, PH, OB, Elliott BM, Robison JG and Cole DJ (1998*b*) Efficacy of reverse transcriptase-polymerase chain reaction screening for micrometastic disease in axillary lymph nodes of breast cancer patients. *Am Surg* **64**: 539–543; discussion 543–544

Lopez-Guerrero JA, Bolufer-Gilabert P, Sanz-Alonso M, Barragan-Gonzalez E, Palau-Perez J, De la Rubia-Comos J, Sempere-Talens A and Bonanad-Boix S (1997) Minimal illegitimate levels of cytokeratin K19 expression in mononucleated blood cells detected by a reverse transcription PCR method (RT-PCR). *Clin Chim Acta* **263**: 105–116

Manni A, Santen RJ, Boucher AE, Lipton A, Harvey H, Drago J, Rohner T, Haagensen D, Glode M and Santner SJ (1984) Evaluation of CEA and GCDFP-15 plasma level during hormonally induced cancer stimulation. *Anticancer Res* **4**: 141–144

Mazoujian G, Bodian C, Haagensen DE, Jr and Haagensen CD (1989) Expression of GCDFP-15 in breast carcinomas. Relationship to pathologic and clinical factors. *Cancer* **63**: 2156–2161

Mazoujian G, Pinkus GS, Davis S and Haagensen DE, Jr (1983) Immunohistochemistry of a gross cystic disease fluid protein (GCDFP-15) of the breast. A marker of apocrine epithelium and breast carcinomas with apocrine features. *Am J Pathol* **110**: 105–112

McGuckin MA, Cummings MC, Walsh MD, Hohn BG, Bennett IC and Wright RG (1996) Occult axillary node metastases in breast cancer: their detection and prognostic significance. *Br J Cancer* **73**: 88–95

Min CJ, Tafra L and Verbanac KM (1998) Identification of superior markers for polymerase chain reaction detection of breast cancer metastases in sentinel lymph nodes [In Process Citation]. *Cancer Res* **58**: 4581–4584

Monteagudo C, Merino MJ, LaPorte N and Neumann RD (1991) Value of gross cystic disease fluid protein-15 in distinguishing metastatic breast carcinomas among poorly differentiated neoplasms involving the ovary. *Hum Pathol* **22**: 368–372

Mori M, Mimori K, Ueo H, Karimine N, Barnard GF, Sugimachi K and Akiyoshi T (1996) Molecular detection of circulating solid carcinoma cells in the peripheral blood: the concept of early systemic disease. *Int J Cancer* **68**: 739–743

Murphy LC, Lee-Wing M, Goldenberg GJ and Shiu RP (1987*a*) Expression of the gene encoding a prolactin-inducible protein by human breast cancers in vivo: correlation with steroid receptor status. *Cancer Res* **47**: 4160–4164

Murphy LC, Tsuyuki D, Myal Y and Shiu RP (1987*b*) Isolation and sequencing of a cDNA clone for a prolactin-inducible protein (PIP). Regulation of PIP gene expression in the human breast cancer cell line, T-47D. *J Biol Chem* **262**: 15236–15241

Noguchi S, Aihara T, Nakamori S, Motomura K, Inaji H, Imaoka S and Koyama H (1994) The detection of breast carcinoma micrometastases in axillary lymph nodes by means of reverse transcriptase-polymerase chain reaction. *Cancer* **74**: 1595–1600

Noguchi S, Aihara T, Motomura K, Inaji H, Imaoka S and Koyama H (1996) Detection of breast cancer micrometastases in axillary lymph nodes by means of reverse transcriptase-polymerase chain reaction. Comparison between MUC1 mRNA and keratin 19 mRNA amplification. *Am J Pathol* **148**: 649–656

Ormsby AH, Snow JL, Su WP and Goellner JR (1995) Diagnostic immunohistochemistry of cutaneous metastatic breast carcinoma: a statistical analysis of the utility of gross cystic disease fluid protein-15 and estrogen receptor protein. *J Am Acad Dermatol* **32**: 711–716

Pagani A, Sapino A, Eusebi V, Bergnolo P and Bussolati G (1994) PIP/GCDFP-15 gene expression and apocrine differentiation in carcinomas of the breast. *Virchows Arch* **425**: 459–465

Pelkey TJ, Frierson HF Jr and Bruns DE (1996) Molecular and immunological detection of circulating tumor cells and micrometastases from solid tumors. *Clin Chem* **42**: 1369–1381

Raj GV, Moreno JG and Gomella LG (1998) Utilization of polymerase chain reaction technology in the detection of solid tumors. *Cancer* **82**: 1419–1442

Schoenfeld A, Kruger KH, Gomm J, Sinnett HD, Gazet JC, Sacks N, Bender HG, Luqmani Y and Coombes RC (1997) The detection of micrometastases in the peripheral blood and bone marrow of patients with breast cancer using immunohistochemistry and reverse transcriptase polymerase chain reaction for keratin 19. *Eur J Cancer* **33**: 854–861

Soomro S and Shousha S (1992) Monoclonal antibody B72.3 immunostaining of breast carcinoma. Patterns of staining and relationship to mucin content and GCDFP-15 reactivity. *Arch Pathol Lab Med* **116**: 32–35

Tsuchiya A, Sugano K, Kimijima I and Abe R (1996) Immunohistochemical evaluation of lymph node micrometastases from breast cancer. *Acta Oncol* **35**: 425–428

Vary CP, Carmody M, LeBlanc R, Hayes T, Rundell C and Keilson L (1996) Allele-specific hybridization of lipoprotein lipase and factor-V Leiden missense mutations with direct label alkaline phosphatase-conjugated oligonucleotide probes. *Genet Anal* **13**: 59–65

Viacava P, Naccarato AG and Bevilacqua G (1998) Spectrum of GCDFP-15 expression in human fetal and adult normal tissues. *Virchows Arch* **432**: 255–260

Watson PH, Snell L and Parisien M (1996) The NCIC-Manitoba Breast Tumor Bank: a resource for applied cancer research. *Cmaj* **155**: 281–283

Wick MR, Lillemoe TJ, Copland GT, Swanson PE, Manivel JC and Kiang DT (1989) Gross cystic disease fluid protein-15 as a marker for breast cancer: immunohistochemical analysis of 690 human neoplasms and comparison with alpha-lactalbumin. *Hum Pathol* **20**: 281–287

Wick MR, Ockner DM, Mills SE, Ritter JH and Swanson PE (1998) Homologous carcinomas of the breasts, skin, and salivary glands. A histologic and immunohistochemical comparison of ductal mammary carcinoma, ductal sweat gland carcinoma, and salivary duct carcinoma. *Am J Clin Pathol* **109**: 75–84

Zippelius A, Kufer P, Honold G, Kollermann MW, Oberneder R, Schlimok G, Riethmuller G and Pantel K (1997) Limitations of reverse-transcriptase polymerase chain reaction analyses for detection of micrometastatic epithelial cancer cells in bone marrow. *J Clin Oncol* **15**: 2701–2708

 *© 1999 Cancer Research Campaign*
